# Mass spectrometry and renal calculi

**Published:** 2010-05-25

**Authors:** M Penescu, VL Purcarea, I Sisu, E Sisu

**Affiliations:** * ‘Carol Davila’ Hospital of Nephrology, 4 Calea Grivitei Street, RO–010701 BucharestRomania; ** ‘Carol Davila’ University of Medicine and Pharmacy, 37 Dionisie Lupu Street, RO–02002, BucharestRomania; *** Institute of Chemistry of the Romanian Academy in Timisoara, 24 Mihai Viteazul Blvd., RO–300223, TimisoaraRomania; **** ‘Victor Babes’ University of Medicine and Pharmacy, 2 Eftimie Murgu Sq., RO–300041, TimisoaraRomania

## Abstract

The present review represents a concise and complete survey of the literature covering  2004–2009, concerning the mass spectrometric techniques involved in the structural investigation of renal calculi. After a short presentation of the fundamental mass spectrometric techniques (MALDI–TOF, QTOF, MS–MS) as well as hyphenated methods (GC–MS, LC–MS, CE–MS), an extensive study of the urinary proteome analysis as well as the detection and quantification by mass spectrometry of toxins, drugs and metabolites from renal calculi is presented.

## Introduction

Over the past decade, progress in mass spectrometry and its hyphenation with the separation techniques has made these tools essential in life sciences. The use of MS is, however, not yet a routine in many fields where it could influence clinical decisions. While medical research using MS is flourishing, few applications have become part of the standard ‘bedside’ practice. This is partly because the transition of MS from a research tool to a reliable clinical diagnostic platform requires rigorous standardization, spectral quality control and assurance, standard operating procedures for robotic and automatic sample application, and standardized controls to ensure the generation of highly reproducible spectra [1]. In a previous review, we focused on contribution of mass spectrometry in finding and characterizing the protein biomarkers [2]. In this review, we provide an overview of new developments in mass spectrometry methods, cover the most promising technical aspects of different approaches to renal calculi analysis (2005–2010), and examine the inherent technical advantages and limitations.

## Fundamentals of mass spectrometry

**Figure 1:**
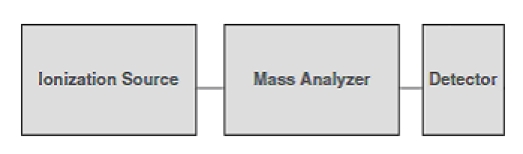
Apical four–chamber recording of a large right atrium and enlarged right ventricular cavity. Diastole before the atrial depolarization. The long masses arise in the posterior and lateral right atrial wall. (asterix)  The tumor extends into the atrial cavity and through the opened tricuspid valve, into the right ventricular inflow.

Mass spectrometry is a sensitive analytical technique, which is able to quantify known analytes and to identify unknown molecules at the picomoles or femtomoles level. A mass spectrometer is an instrument, which volatilizes and ionizes molecules and measures, more precisely ion abundance, as a function of the ionic mass–to–charge ratio. (Figure 1) Mass spectrometers are unable to detect neutral molecules and radicals [3]. Typically, a mass spectrometer is made up of the following components:

- a source to produce ions
- one or several mass analyzers
- a detector to measure the abundance of ions

### Ion Sources

The analyzed samples in the ion sources are ionized prior to analysis in the mass spectrometer. Depending on the nature of the ionization process, and also on the nature of the atoms and molecules themselves, positive and negative ion types can be formed [3,4]. Some ionization techniques are very energetic and cause extensive fragmentation. Other techniques are softer and only produce ions of molecular species. Ion sources exist under two types: liquid-phase ion sources and solid-state ion sources [5].

In liquid–phase ion sources, the analyte is in solution. This solution is introduced, by nebulization, as droplets into the source, where ions are produced at an atmospheric pressure and focused into the mass spectrometer through some vacuum pumping stages. Electrospray (**ESI**), atmospheric pressure chemical ionization (**APCI**) and atmospheric pressure photoionization (**APP**) sources correspond to this type.

In solid–state ion sources, the analyte is in an involatile deposit. This deposit is then irradiated by energetic particles or photons that desorb ions near the surface of the deposit. These ions can be extracted by an electric field and focused towards the analyser. Matrix–assisted laser desorption (**MALDI**), surface–enhanced laser desorption/ionization (**SELDI**), secondary ion mass spectrometry (**SIMS**), plasma desorption (**PD**) and field desorption (**FD**) sources all use this strategy to produce ions. Fast atom bombardment (**FAB**) uses an involatile liquid matrix. The ion sources produce ions mainly by ionizing a neutral molecule in the gas phase through electron ejection, electron capture, protonation, deprotonation, adduct formation or by the transfer of a charged species from a condensed phase to the gas phase.

### Mass analyzers

**Figure 2:**
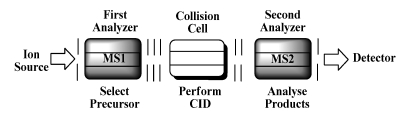
Principle of tandem mass spectrometry (MS/MS)

All mass analyzers perform a separation of ions according to their m/z The simplest way of ion separation is just to let them fly and measure their time of flight. This type of analyzer is called time of flight (**TOF**). Here, electrostatic potential gradients are used to accelerate/decelerate the ions. Another way to ion separation is achieved by the interaction of ions with an electrostatic (electric sector analyzer,** ESA **or orbitrap (**OT**)) or a magnetostatic (magnet,** B**) field. A resonant electromagnetic field is applied in quadrupoles (**Q**), and three–dimensional or linear ion traps (**3D–IT** and **LTQ**, respectively). A combination of electric (E) and magnetic (B) fields is used in Fourier transform ion cyclotron resonance (**FTICR**) instruments and also the technique of accelerator mass spectrometry (**AMS**) [5,6]. Tandem mass spectrometry (**MS/MS**) is a technique where structural information on sample molecules is obtained by using multiple stages of mass selection and mass separation. Tandem mass spectrometry requires the fragmentation of the precursor ion selected by the first analyzer in order to allow the second analyzer to analyze the product ions. This can be achieved by collisional activation via collisions of (selected) ion with neutral gas molecules (collision–induced dissociation, CID). CID is a two–step process: ion translational energy is converted into ion internal energy in the collision event, and subsequently unimolecular decomposition of the excited ion may yield various product ions [5–7].Two stages of mass analysis are required: one to select the precursor ion from other ions generated in the ion source, and one to analyze the product ions after the collisions (Figure 2). 

### Detector 

Modern mass spectrometers are equipped with detectors of great sensitivity. The detectors most commonly used include the electron multiplier, the photomultiplier, the conversion dynode, the Faraday cap, the array detector, and the charge or inductive detector. From the operational point of view, reliable vacuum systems are a prerequisite for mass spectral measurements [7,8].

The final step of a **mass spectral** analysis is the recording of the mass spectrum by detecting the ions after their separation. The mass spectrum is a unique characteristic of a compound. In general, it contains information on the molecular mass of an analyte (molecular ion) and the masses of its structural fragments.

### Hyphenated methods

In order to analyze a complex mixture, for example natural products, a separation technique – gas chromatography (**GC**), liquid chromatography (**LC**) or capillary electrophoresis (**CE**) – is coupled with the mass spectrometer. The separated products must be introduced one after the other into the spectrometer, either in the gaseous state for **GC–MS**, or in solution for **LC–MS** and **CE–MS**. This can occur in two ways: the eluting compound is collected and analyzed off–line; or the chromatograph is directly connected to the mass spectrometer and the mass spectra are acquired while the compounds of the mixture are eluted. The latter method operates on–line. The most obvious advantage drawn from the coupling of a separation technique with a mass spectrometry consists in obtaining a spectrum used in identifying the isolated product and evaluating the components quantitatively [6,8].

## Mass spectrometry in Urinary Proteome Analysis

Proteome research related to nephrology has generally focused on the examination of urine because it is easily accessible in a large quantity without the use of invasive procedures. Furthermore, as a rule, pathophysiologic changes in the genitourinary tract and the kidney are reflected by changes in the urinary proteome [9]. Although many studies have shown that proteins in biologic fluids may degrade rapidly when handled inappropriately, urinary proteins have been shown to remain stable long enough to perform reliable proteome analysis [10]. The reliable identification of protein expression patterns in various renal calculi that differentiate the diseased kidneys from the healthy ones or those that distinguish between different stages of a disease have now started to become feasible.

Proteomic analysis of renal calculi indicates an important role for inflammatory processes in calcium stone formation. Lederer and all [11] analyzed isolated renal stone matrix proteins with 1D RPLC–MS, MALDI–TOF MS and immunohistochemical methods identifying 158 proteins with high confidence, including 28 common proteins. Several investigators have examined the protein content of CaOx stones, identifying proteins either coating the crystals or embedded in the stones’ crystalline structure, such as OPN, THP, and urinary PF1. The significance of these differences in protein association with stones and their roles in inhibition, promotion, or reaction to stone formation, while heavily investigated, remain to be thoroughly defined. In addition to the contribution of constitutive urinary proteins, the elicited inflammatory responses may further contribute to the proteomic milieu. These proteins, whether from cellular compartments of the kidney or from filtered serum proteins, will all likely contribute to the composition of the stone proteome. The results of the group identified previously, unreported stone matrix proteins (SMP), some of which may be involved in critical elements of stone formation. In conclusion, the proteomic analysis of a limited set of renal stones has significantly expanded the list of known SMP. The diverse origin of these proteins (i.e., extracellular, intracellular, membrane proteins) attests a complex and multifactorial pathogenesis of stone formation. The data do not provide discrete mechanistic information regarding the stones’ formation. Indeed, a possible significant portion of the identified SMP may be derived from the cellular damage resulting from stone growth and may not involve directly with stone growth per se [11].

It is known that urinary calculi are often complicated in patients with gout, hyperuricemia, or hypouricemia. In order to examine the growth mechanism of urinary calculi in these patients and to prevent recurrence of calculi, it is important to carefully analyze each patient's individual pathological calculus. Although not generally known, several proteins, collectively dubbed ‘the matrix,’ have been reported as organic components in renal stones (first, albumin then alpha-globulins and gamma-globulins followed). Protein Z, a vitamin K–dependent plasma protein, has been firstly detected in a renal calculus, along with osteopontin and prothrombin, by Fujimori group [12]. The renal calculus was obtained from a hyperuricemic patient. Several proteins from the renal calculus, which have migrated in 2D–PAGE, were excised from the gel, digested with trypsin in gel, and then applied to LC–MS.  The proteins from each isolated spot in 2D gel were analyzed by tandem mass spectrometry; many MS/MS spectra were obtained. The computerized protein identification program, SEQUEST, a tandem mass spectrum database–matching tool, was applied. Three proteins, osteopontin, prothrombin, and protein Z, were identified in this renal stone. The spectrometer was equipped with a nanoelectrospray interface and an ion trap. Four peptides were determined from  protein  Z (the positions were 203–215, 232–240, 320–327, and 413–418 amino acids, respectively) in the calculus, through LC–MS/MS analysis. Authentic protein Z was also analyzed by using the same method, and all four peptides determined in the calculus were similarly identified. Whereas protein Z has been reported to be one of the vitamin K–dependent calcium–binding proteins, its role has not been well established yet. Because the physiological role of protein Z is hardly understood, the fact that it has been identified in a renal calculus is of great interest. This finding should prove helpful in any future examination of the role of protein Z in the body and the lithic generation mechanism in patients with urolithiasis.[12].

The kidney stone formation is a complex process involving multiple factors. Kidney stones invariably comprise a combination of inorganic crystals and organic macromolecules consisting principally of proteins. Many proteins occur in stones, but their role in urolithiasis remains unknown. Calculi contain some proteins normally present in urine, in addition to others arising from injury inflicted by the stones themselves, making it impossible to discriminate between those that bind to the stone as it grows, but play no role in its development, and those that may be involved in regulating the formation of stone crystals. Tandon and his coworkers were able to fractionate renal stone matrix proteins, to purify most potent antilithiatic protein, to study its effect on calcium oxalate crystal growth system and to characterize them by using mass spectrometry [13]. Proteins having MW>10 kDa were subjected to anion exchange and molecular–sieve chromatography. Protein fractions were tested for their effects on CaOx crystal growth. Most potent fraction P2′ was excised, in–gel tryptic digested and identified by matrix assisted laser desorption/ionization–time of flight (MALDI–TOF) MS. As a result, an anionic protein (MW aprox. 42 kDa) with potent inhibitory activity against CaOx crystal growth was purified. Its homogeneity was confirmed by RP–HPLC. It was identified by MALDI–TOF–MS followed by database search on MASCOT server as human phosphate cytidylyltransferase 1, beta. The molecular weight of this novel CaOx crystal growth inhibitor, from human renal stone matrix, is also the same as that of human phosphate cytidylyltransferase 1, choline [13].

Of all types of renal stones, calcium oxalate (CaOx) is the most common composition found by chemical analysis. Up until present, the pathogenic mechanisms of stone formation remain unclear. One long–standing hypothesis is that stone formation is related to intratubular crystal nucleation, growth, and aggregation. The urine from patients with nephrolithiasis is commonly supersaturated with calcium and oxalate ions, favoring CaOx crystal nucleation and growth.  Additionally, there are urinary substances known as ‘stone inhibitors’ in the normal renal tubular fluid that inhibit intratubular crystal growth, aggregation, and/or adhesion to renal epithelial surfaces. These substances include proteins, lipids, glycosaminoglycans, and inorganic compounds. Abnormality in function and/or expression levels of these molecules, especially proteins, in the urine and renal tubular fluid has been proposed to be associated with stone formation. The identification of additional stone inhibitors would increase the understanding of the pathogenesis and pathophysiology of nephrolithiasis. Thongboonkerd and his coworker [14] have combined conventional biochemical methods with recent advances in mass spectrometry (MS) to identify a novel calcium oxalate (CaOx) crystal growth inhibitor in normal human urine. Anionic proteins were isolated by DEAE adsorption and separated by HiLoad 16/60 Superdex 75 gel filtration. A fraction with potent inhibitory activity against CaOx crystal growth was isolated and purified by anion exchange chromatography. The protein in two subfractions that retained inhibitory activity was identified by MS (MALDI–TOF) matrix–assisted laser desorption/ionization–time–of–flight and MS (ESI–QTOF–MS/MS) electrospray ionization–quadrupole–time–of–flight tandem, as human trefoil factor 1 (TFF1). Western blot analysis confirmed the mass spectrometric protein identification. Functional studies of urinary TFF1 demonstrated that its inhibitory potency was similar to that of nephrocalcin. The inhibitory activity of urinary TFF1 was dosed dependently and was inhibited by TFF1 antisera. Anti–C–terminal antibody was particularly effective, consistent with proposed model, in which the 4 C–terminal glutamic residues of TFF1, interact with calcium ions to prevent CaOx crystal growth. Concentrations and relative amounts of TFF1 in the urine of patients with idiopathic CaOx kidney stone were significantly less (2.5–fold for the concentrations and 5– to 22–fold for the relative amounts) than those found in controls. These data indicate that TFF1 is a novel potent CaOx crystal growth inhibitor with a potential pathophysiological role in nephrolithiasis [14]

Adult human urine is often supersaturated with calcium and oxalate ions, which can lead to the precipitation of calcium oxalate (CaOx) crystals thereby making individuals prone to stone formation. Proteins are found as a major component in the matrix and organic matrix and are considered to have a potential role in stone formation, growth and crystal–membrane interaction. Proteins, which have crystal binding affinity, could play a critical role in the mediation of the earliest events in kidney stone formation. Shafqat and his group [15] characterized proteins from the inner core and outer matrix of calcium oxalate (CaOx) renal stones, in order to understand the mechanism of stone genesis. Inner core and outer matrix of CaOx renal stones were separated and proteins were extracted with a buffer containing SDS and beta–mercaptoethanol. The proteins were analyzed and purified by SDS–PAGE and RP–HPLC respectively. The protein bands from gel and protein fractions were sequenced by MALDI TOF mass spectrometry. ELISA, western and slot blot immunoassays were performed to confirm the identity of the proteins in stones and urine of the stone formers. The potential of the identified protein as an effective promoter or inhibitor was assessed by observing their effects on CaOx crystallization by using an aggregometer. The inner core extract predominantly exhibited protein species in the molecular weight range of 12–14 kDa. However, a 66 kDa band, identified as osteopontin was also detected in the inner core along with outer matrix and in the urine of stone formers and non stone formers. The purification of low molecular weight proteins was carried out by reversed phase HPLC. Tandem mass spectrometry analysis identified them as myeloperoxidase chain A (MPO–A), alpha–defensin, and calgranulin. ELISA, western blot and slot–blot immuno–assays further confirmed their presence restricted to the inner core and not in the outer matrix. Turbidity assays showed that low molecular weight renal stone proteins promoted the aggregation of CaOx crystals. From these results, the author concluded that persistent hyperoxaluria leads to tubular epithelial injury, resulting in the release of these anti-inflammatory proteins. These proteins could have been firstly adsorbed on CaOx crystals, so, they became a part of nucleation process leading to inner matrix formation [15].

OPN (osteopontin) is a highly phosphorylated glycoprotein present in many tissues and body fluids. In urine, OPN is a potent inhibitor of nucleation, growth and aggregation of calcium oxalate crystals, suggesting that it has a role in the prevention of renal stone formation. The role of OPN in nephrolithiasis is, however, somewhat unclear, as it may also be involved in urinary stone formation, and it has been identified among the major protein components of renal calculi. Most likely, the function of OPN in urine is dependent on the highly anionic character of the protein. Besides a very high content of aspartic and glutamic residues, OPN is subjected to significant PTM (posttranslational modification), such as phosphorylation, sulfation and glycosylation, which may function as regulatory switches in promotion or inhibition of mineralization. Sorensen and all [16] have characterized the PTMs of intact human urinary OPN and N–terminal fragments thereof. Urine samples from seven healthy donors, with normal renal function and no history of urinary disease, were analyzed by Western blotting, to examine the molecular forms of OPN in urine. OPN was purified from human urine by anion exchange followed by RP–HPLC separation. The sequencing of the OPN–containing fraction showed two sequences: a major, and a minor one, both corresponding to the N–terminal part of human OPN. SDS/PAGE revealed the presence of three bands migrating at approx. 45 (OPN45), 50 (OPN50) and 60 kDa (OPN60) respectively, and Western blotting confirmed their OPN nature. The masses of the OPNs were determined by linear MALDI–TOF–MS. MS analysis showed a mass of approx. 37.7 kDa for OPN60, whereas the sample containing OPN45 and OPN50 showed one broad mass peak with an average value of approx. 29.3 kDa. In order to estimate the total number of phosphate groups present, OPN60 and the fragments OPN45/OPN50 were treated with ALP. The molecular mass of dephosphorylated OPN60 was of 37.1 kDa, corresponding to a loss of approximately eight phosphate groups. Phosphatase treatment of the N–terminal OPN fragments, reduced the mass to 28.8 kDa, corresponding to a loss of approximately six phosphorylations. The subtraction of the theoretical mass of the human OPN polypeptide (33714 Da) from the observed average mass of dephosphorylated OPN60 (37.1 kDa), leaves approx. 3.4 kDa, which means that the other PTMs must be accounted for. In addition, one sulfated tyrosine and five O–linked glycosylations were identified in OPN, whereas no N–linked glycans were detected. Peptide mapping and immunoblotting using different monoclonal antibodies showed that the N–terminal fragments present in urine are generated by proteolytic cleavage at Arg_228_–Leu_229_ and Tyr_230_–Lys_231_[16]

The interaction between crystals and renal tubular cells has been proposed to be a crucial event that elicits subsequent cellular responses, leading to kidney stone formation. Nevertheless, the molecular mechanisms of these cellular responses remain poorly understood. Thongboonkerd  and coworkers [17] performed a gel–based differential proteomics study to examine cellular responses (as determined by altered protein expression) in Madin–Darby canine kidney (MDCK) cells, which were derived from dog kidney and exhibited distal renal tubule phenotype, during calcium oxalate dehydrate (COD) crystal adhesion. MDCK cells were grown in a medium with or without COD crystals (100 micro g/ml) for 48 h. Crystal adhesion was illustrated by phase–contrast and scanning electron microscopy (SEM). Flow cytometry using annexin V/propidium double staining iodide showed that the percentage of cell death did not significantly differ between cells with and without COD crystal adhesion. Cellular proteins were then extracted, resolved with two–dimensional gel electrophoresis (2–DE), and visualized by SYPRO Ruby staining (*n* ) 5 gels per group). Quantitative intensity analysis revealed 11 significantly altered proteins, 10 of which were successfully identified by quadrupole time–of–flight (Q–TOF–MS), peptide mass finger printing and/or tandem MS (MS/MS): Lamin B, Heterogeneous nuclear ribonucleoprotein H1, Cytokeratin 7, Ornithine aminotransferase (OAT –mitochondrial precursor isoform), Branched –chain–amino–acid aminotransferase, Alcohol dehydrogenase (ADH), Annexin A2 (Anx Ⅱ), Glyceraldehyde–3–phosphate dehydrogenase (G3PDH), Galactose–specific lectin. An increase in annexin Ⅱ was confirmed by 2–D Western blot analysis. The data from their experiments may lead to better understanding of the cellular responses in distal renal tubular cells during COD crystal adhesion [17].

The matrix stones are rare calculi that present not as crystalline solids but as soft, proteinaceous material within the kidney collecting system. The converse of stone matrix and matrix stones have little minerals and they are primarily composed of organic material that could be ideal for protein extraction. They have been identified in patients with recurrent urinary tract infections and in proteinuric patients with glomerulonephritis and end–stage kidney disease on hemodialysis. In an attempt to understand the factors responsible for matrix stone formation, Canales and all [18] determined the mineral, topographical, and proteomic composition of a surgically extracted matrix stone using modern identification technology. Following wide–angle X–ray diffraction (XRD) and scanning electron microscopy (SEM), they homogenized a surgically removed matrix stone, extracted and purified protein, and analyzed samples by using tandem mass spectrometry for proteomic composition. Resulting spectra were searched by using ProteinPilot 2.0, and the identified proteins were reported with >95% confidence. Primary XRD mineral analysis was a biological apatite, and SEM revealed fibrous, net–like laminations containing bacterial, cellular, and crystalline material. Out of the 33 unique proteins identified (among them: Protein C, Prothrombin  Anti–TNFalpha–antibody, Calgranulin A, Calgranulin B, Cathepsin G, Ig light chain, IgG2 IgG heavy chain, Ig kappa light chain, Lactoferrin, Lysozyme C,Myeloblastin, Myeloperoxidase, Albumin, Histidine–rich glycoprotein, Plasminogen, Hemoglobin alpha chain, Hemoglobin beta chain), 90% have not been previously reported within matrix stones, and over 70% may be considered inflammatory or defensive in nature. The characterization of other matrix stone proteomes, in particular from non–infectious populations, may yield insights into the pathogenesis of this rare stone, as well as the mineralogical process that occurs within crystalline calculi [18].

## Detection and quantification of toxins, drugs and metabolites from renal calculi, by mass spectrometry

Oxalic acid is a major product of ascorbic acid oxidation, having the potential to crystallize like calcium oxalate in the urinary space. Huge oral doses of ascorbic acid modestly increase urinary oxalic acid excretion and could theoretically increase the risk of stone formation in susceptible people. The rate limitation of intestinal ascorbic acid transport makes it unlikely that the oral doses higher than 500 mg/d will increase oxalic acid excretion and stone risk, proportionately with the intravenous administration that bypasses this barrier. Hoffer and all [19] developed gas chromatography mass spectrometry (GC–Q–MS) methodology, sampling and storage procedures for oxalic acid analysis without the interference of the ascorbic acid and measured urinary oxalic acid excretion, in people who were administered intravenous ascorbic acid in doses ranging from 0.2 to 1.5 g/kg according to body weight. In vitro oxidation of the ascorbic acid to oxalic acid did not occur when urine samples were immediately brought to a pH level of less than 2 and stored at –30 degrees C for 6 hours [19].

Several antiretroviral drugs such as nelfinavir, efavirenz and atazanavir have been presented to cause urinary stone [20–22]; Strebel and all [23] reported the first case of pro–lithogenic amprenavir – in a female with HIV infection – adding this to the growing list of antiretroviral drugs associated with urinary stones. The abdominal CT of the patient revealed three obstructing stones (of 4x8mm; 2x1cm; 4 mm in diameter). Before undergoing an interventional treatment, one stone had passed spontaneously. The stone was analyzed by liquid chromatography with mass spectrometry (LC–MS). The analysis of the stone revealed a composition of 95% amprenavir and 5% ritonavir. It was the first time a report was made on a urinary stone composed of unmodified amprenavir [23].

An unprecedented epidemic of renal disease affecting children after the consumption of melamine–tainted milk products (MTMP) was reported two years ago. The spectrum of this disease, its clinical features, and criteria, for laboratory diagnosis and monitoring, have not been very well defined. Urolithiasis is an uncommon condition in children and therefore, it was observed that this issue unexpectedly increased the incidence of urinary stones and renal failure in infants, in China, before 2008. The association between this disease and the exposure to MTMP has not yet been defined by epidemiological studies. Although melamine alone has a low animal toxicity and its being rapidly eliminated, unchanged after ingestion, led to the conclusion that it can form an insoluble complex with cyanuric acid, a structural analogue of melamine which often co–exists as either an impurity or metabolite, thereby causing crystalluria, kidney stones and nephrotoxicity. The pet foods were found to contain melamine and cyanuric acid in high concentrations, which were traced to wheat gluten and rice protein sourced from China. In addition, the diagnosis of melamine–associated renal stone disease (MARSD) is suspected due to the presence of urinary tract stones on ultrasound examination in patients exposed to MTMP. As melamine associated renal stones (MARS) can be spontaneously passed out of the urinary tract, Tam and all [24] developed a non-invasive method for the diagnosis and monitoring of MARSD patients. For the detection and quantification of urinary melamine, they used an ESI–TQ–MS (triple–quadruple tandem mass spectrometer) operating in a positive mode with pneumatically assisted electrospray. A GC–MS (gas–chromatography mass–spectrometer) was employed for the measurement of urinary cyanuric acid. They found a strong correlation between renal stone size and urinary melamine concentration. For stones with the diameter of less than 10 mm, a 10 micro g/mmol creatinine increase in the urinary melamine concentration is associated with the approximately 1 mm increase in the size of the stone. The high degree of the correlation strongly suggests that melamine is related to stone formation in humans. Using receiver operating characteristic analysis, they proposed that patients, who have a persistent melamine level above the optimal cut–off value of 7.1 micro g melamine/mmol creatinine in urine, might have a significant exposure of melamine–tainted products. Unlike melamine, urinary cyanuric acid is not significantly different between cases and controls. Pathophysiological findings from feeding animals with melamine and cyanuric acid may not be directly applicable to humans. Finally, they found out that both melamine and urine metabolic lithogenic factors are important in the formation of melamine–related stones. Apart from aiding in the case screening and confirmation, the urine melamine level might as well be an indicator of residual melamine load in the body and thus, it is useful for the following–up and monitoring of the confirmed cases [24]. 

**Figure 3:**
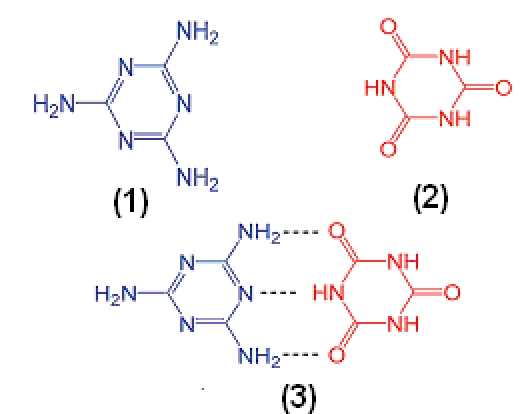
Chemical structure of melamine (1), cyanuric acid (2), and melamine cyanurate (3).

The outbreak of food adulteration with melamine was firstly discovered in dairy products, and this adulterant is now found in other types of food products such as eggs and seafood. In 2007, pet food adulteration with melamine leading to kidney toxicity in cats and dogs was reported. It is believed that melamine per se is nontoxic. However, when it encounters cyanuric acid, an analogue of melamine, a stable compound (melamine cyanurate), as shown in Figure Figure 3, a poor aqueous solubility is formed [25]. This compound precipitates in renal tubules, results in the formation of kidney stones, and eventually leads to renal toxicity. Matrix–assisted laser desorption/ionization mass spectrometry (MALDI–MS) was applied by Che and colab. [26] to the direct analysis of melamine cyanurate (MC). The three commonly used MALDI matrixes, namely, cyano–4–hydroxycinnamic acid (CHCA), sinapinic acid (SA), and 2,5–dihydroxybenzoic acid (DHB), were able to desorb/ionize melamine from MC upon N2 laser irradiation, with CHCA showing the highest detection sensitivity in the positive mode. Only DHB and SA were able to desorb/ionize cyanuric acid from MC in the negative mode but with a remarkably lower sensitivity.

The urine matrix resulted in ion peaks interfering and it suppressed the ion intensity of melamine, while, a cleanup process consisting of a simple wash–up, eliminated such interference and enhanced the ion intensity. The procedure developed by the authors allowed the detection of melamine from a small amount of melamine cyanurate, down to as low as 12.5 micro g in 1 mL of urine, and could be used for the screening analysis of urine residue samples. In combination with accurate mass measurement and tandem mass spectrometric analysis, the method could allow the confirmatory identification of the analyte. In terms of practical application, the method was first applied in the detection of melamine, from urine stone/residue samples, collected from patients clinically confirmed as having kidney stones associated with the consumption of melamine–tainted food products. The advantages of this method are the simplicity of sample preparation and the rapidity of MALDI–MS analysis. The analytical time per sample, is less than 7 min when the method is applied in a high–throughput manner. This method allows the specific detection of the chemical composition of solid urine stone/residue samples, and has a potential for use in the clinical diagnosis of other complex biological samples, in the solid state [26].

Uric acid (2,6,8–trihydroxypurine, UA) is the major nitrogenous compound in urine, but it is also found in other biological fluids such as serum, blood and saliva. Around 8% of renal calculi are formed by UA. It has also been demonstrated that a group of calcium oxalate monohydrate renal calculi have a core formed by important amounts of UA, which act as heterogeneous nucleant. In some cases, due to the little size of the core, or the presence of an abundant organic matter, the presence of uric acid cannot be confirmed, in spite of its importance to establish the calculus etiology, and, therefore, the most appropriate pharmacologic and/or dietetic treatment. Elevated levels of UA can be caused by many factors, including increased alcohol consumption, obesity, diabetes, high cholesterol, high blood pressure, kidney disease, and heart disease. UA is the main final product of purine metabolism and its determination in urine is a powerful indicator of metabolic alterations or disease appearance. Grases and all proposed [27] a very simple and direct method for the determination of uric acid, in various biological matrices, based on high–performance liquid chromatography and mass spectrometry (HPLC–MS). Chromatographic separations were performed with a stationary phase Zorbax Sax Column, an anion exchange resin, with 50% sodium citrate 1mM at pH 6.5 and 50% acetonitrile, as a mobile phase delivered at a flow rate of 1 ml/min. The detector counted negative ions by monitoring m/z 167.1, which corresponds to the urate anion. The method does not use an internal standard but quality control samples were used. Recovery tests of added standard have been successfully performed in urine and saliva samples, thus showing an appropriate accuracy of the method. The limit of quantization found was of 70 mg/l. Different urine and saliva samples were analyzed and applied to the determination of trace amounts of uric acid in the core of some selected calcium oxalate renal calculi [27]. 

Urinary stones may form anywhere in the renal tract and will often pass through in an uneventful fashion. Recurrence of stone disease is a problem and it is recognized that, without medical treatment, 50% of patients will form another stone within 10 years. The determination of citrate excretion is important in the investigation of patients with recurrent stone disease, because the formation of calcium–based stones, which accounts for 85% of all stones formed in the urinary tract, is known to be inhibited by citrate. Citrate in urine inhibits the formation of calcium salts by forming soluble complexes with calcium, and, hypocitraturia is associated with an increased risk of stone formation. Hypocitraturia, defined as a citrate excretion of <1700 mmol/24 h, is found in renal tubular acidosis, chronic diarrhea, chronic diuretic use and chronic dehydration. In these situations, supplementation with potassium citrate is the best treatment, either as effervescent or wax–coated tablets. The measurement of urine citrate is important, being requested by urologists, to assess the risk of further stone formation and the benefit of the treatment. Kavanag and his group [28] developed a simple and rapid liquid chromatography tandem mass spectrometry (LC–MS/ MS) method, for the analysis of urinary citrate, and compared it with a current enzymatic assay. For the LC–MS/MS assay, samples were prepared in a deep–well block by adding 10 mL of urine and 20 mL of internal standard to 400 mL of water. After the mixing, 3 mL of the diluted sample was injected into the LC–MS/MS system. A LC system was used to isocratically elute a C18 column (50_2.1 mm) with 0.4 mL/min water, containing 2 mmol/L ammonium acetate and 0.1% (v/v) formic acid. A step gradient of 100% methanol containing 2 mmol/L ammonium acetate and 0.1% (v/v) formic acid was used to wash the column. The retention times were 1.4 min for citrate and 1.4 min for d4–citrate. The cycle time was of 4.0 min, injection to injection. The analytes were monitored by using a tandem mass spectrometer, which operated in multiple reaction monitoring mode by using the following transitions, citrate m/z 191.00>111.0 and d4–citrate m/z 195.0>113.0 [28].

Many clinical and epidemiological studies relate hyperuricosuria to calcium oxalate stone formation. Uric acid crystals have crystallographic features similar to calcium oxalate monohydrate (COM) crystals, and for this reason, they act as active inducers of COM heterogeneous nucleation. Consequently, this fact would explain the physicochemical mechanism by which uric acid affects calcium oxalate crystallization. In spite of these facts, calcium oxalate/uric acid mixed calculi are not very common, corresponding to only 2.6% of all renal calculi. Costa–Bauza and all [29] evaluated the presence of uric acid in the beginning zone of different types of ‘pure’ calcium oxalate renal calculi with the aim of establishing the degree of participation of uric acid crystals in the formation of such calculi. For this, the core or fragment of different types of ‘pure’ calcium oxalate renal calculi was detached, pulverized and uric acid extracted. Uric acid was determined by using HPLC–MS (high–performance liquid chromatography/mass spectrometry). Chromatographic separations were performed on an anion exchange resin. Mass spectral identification of uric acid was carried out with an electrospray ionization interface and a quadrupole mass analyzer. The mobile phase was nebulized by nitrogen gas at 350 degrees C, with a flow rate of 13 L/min, into an electrospray mass analyzer. The detector counted negative ions with selected ion monitor (SIM) mode, by monitoring m/z = 167.1, which corresponds to the urate anion, the most abundant ion. If the papillary calculi with a core, are constituted by COM crystals and organic matter in calcium oxalate monohydrate (COM), then the concentration of uric acid is of 0.030 ± 0.007%. Moreover, 0.031 ± 0.008% uric acid was found in COM papillary calculi with a core constituted by hydroxyapatite. 0.24 ± 0.09% uric acid was found in COM unattached calculi (formed in renal cavities) with the core mainly formed by COM crystals and organic matter. 20.8 ± 7.8% uric acid was found in COM unattached calculi with the core formed by uric acid identifiable by scanning electron microscopy (SEM), coupled to X–ray microanalysis. 0.012 ± 0.004% uric acid was found in calcium oxalate dihydrate (COD) unattached calculi containing little amounts of organic matter. 0.0030 ± 0.0004% of uric acid was found in COD unattached calculi containing little amounts of organic matter and hydroxyapatite. From these results, the authors demonstrated that uric acid can play an important role as inducer (heterogeneous nucleant) of COM unattached calculi, with the core formed by uric acid identifiable by SEM, coupled to X–ray microanalysis (these calculi constitute the 1.2% of all calculi), as well as in COM unattached calculi, with the core mainly formed by COM crystals and organic matter (these calculi constitute the 10.8% of all calculi) [29].

## References

[1] Petricoin EF, Liotta LA (2004). Proteomic approaches in cancer risk and response assessments. Trends Mol. Med..

[2] Penescu M, Sisu I, Purcarea VL, Sisu E (2009). The applications of mass spectrometry  for identifying modern biochemical markers of nephropathies. Farmacia.

[3] Wanner KT, Hofner G (2007). Mass Spectrometry in Medicinal Chemistry.

[4] Vekey K, Telekes A, Vertes A (2008). Medical applications of mass spectrometry.

[5] Hoffmann E, Stroobant V (2007). Mass Spectrometry –Principles and Applications.

[6] Niessen WMA (2006). Liquid Chromatography–Mass Spectrometry.

[7] Downard K (2004). Mass Spectrometry. A Foundation Course.

[8] Ardrey RE (2003). Liquid Chromatography–Mass Spectrometry: an introduction.

[9] Fliser  D, Novak  J, Thongboonkerd  V, Argiles A, Jankowski V, Girolami MA, Jankowski J (2007). Advances in Urinary Proteome Analysis and Biomarker Discovery. J Am Soc Nephrol.

[10] Theodorescu D, Wittke S, Ross  MM, Walden M (2006). Discovery and validation of new protein biomarkers for urothelial cancer: A prospective analysis. Lancet Oncol.

[11] Merchant ML, Cummins TD, Wilkey DW, Salyer  SA, Powell DW (2008). Proteomic analysis of renal calculi indicates an important role for inflammatory processes in calcium stone formation. Am J Physiol Renal Physiol..

[12] Kiyoko K, Tomoyo  Y, Kazuya N, Kenichi M, Maki O (2004). Detection of protein Z in a renal calculus composed of calcium oxalate monohydrate with the use of liquid chromatography–mass spectrometry/mass spectrometry following two–dimensional polyacrylamide gel electrophoresis separation. Anal.Biochem.

[13] Priyadarshini, Singh SK, Tandon  C (2009). Mass spectrometric identification of human phosphate cytidylyltransferase 1 as a novel calcium oxalate crystal growth inhibitor purified from human renal stone matrix. Clin.Chim.Acta..

[14] Chutipongtanate S, Nakagawa Y, Sritippayawan S (2005). Identification of human urinary trefoil factor 1 as a novel calcium oxalate crystal growth inhibitor. J. Clin. Invest.

[15] Mushtaq S, Siddiqui AA, Naqvi ZA (2007). Identification of myeloperoxidase, alpha defensin and calgranulin in calcium oxalate renal stones. Clin.Chim.Acta..

[16] Christensen B, Petersen TE, Sorensen ES (2008). Post–translational modification and proteolytic processing of urinary osteopontin.. Biochem. J.

[17] Semangoen T, Sinchaikul S, Chen ST (2008). Altered Proteins in MDCK Renal Tubular Cells in Response to Calcium Oxalate Dehydrate Crystal Adhesion: A Proteomics Approach. J. Proteom. Res.

[18] Canales BK, Anderson L, Higgins L (2009). Proteomic analysis of a matrix stone: a case report. Urol. Res..

[19] Robitaille L, Mamer OA, Miller  WH (2009). Oxalic acid excretion after intravenous ascorbic acid administration. Metabol. Clin.Experimental..

[20] Engeler DS, Wyler S, Neyer M (2008). Feasibility of early intravesical instillation chemotherapy after transurethral resection of the bladder: a prospective evaluation in a consecutive series of 210 cases. Scand J Urol Nephrol.

[21] Wirth GJ, Teuscher J, Graf JD (2006). Efavirenz induced urolithiasis. Urol.Res..

[22] Chang HR, Pella PM (2006). Atazanavir urolithiasis. N Engl J Med.

[23] Feicke A, Rentsch KM, Oertle D (2008). Same patient, new stone composition: amprenavir urinary stone.. Antivir Ther..

[24] Lam CW, Lan L, Che X (2009). Diagnosis and spectrum of melamine–related renal disease: Plausible mechanism of stone formation in humans. Clin.Chim.Acta..

[25] Dobson RLM, Motlagh S, Quijano M (2008). Identification and characterization of toxicity of contaminants in pet food leading to an outbreak of renal toxicity in cats and dogs.. Toxicol. Sci.

[26] Tang  HW, Ng KM, Chui   SS (2009). Analysis of Melamine Cyanurate in Urine Using Matrix–Assisted Laser Desorption /Ionization Mass Spectrometry. Anal. Chem..

[27] Perello J, Sanchis P, Grases F (2005). Determination of uric acid in urine, saliva and calcium oxalate renal calculi by high–performance liquid chromatography/mass spectrometry.. J. Chromatogr. B.

[28] Keevil BG, Owen L, Thornton S (2005). Measurement of citrate in urine using liquid chromatography tandem mass spectrometry: comparison with an enzymatic method. Ann Clin Biochem.

[29] Grases F, Sanchis P, Perello J (2006). Role of uric acid in different types of calcium oxalate renal calculi. Int.J.Urol..

